# Sesquiterpenoids and Diterpenoids from the Wood of *Cunninghamia konishii* and Their Inhibitory Activities against NO Production

**DOI:** 10.3390/molecules21040490

**Published:** 2016-04-13

**Authors:** Chi-I Chang, Chien-Chih Chen, Che-Yi Chao, Sheng-Yang Wang, Hsun-Shuo Chang, Ping-Jyun Sung, Guan-Jhong Huang, Yen-Cheng Li, Yueh-Hsiung Kuo

**Affiliations:** 1 Department of Biological Science and Technology, National Pingtung University of Science and Technology, Pingtung 912, Taiwan; changchii@mail.npust.edu.tw; 2 Department of Biotechnology, Hungkuang University, Taichung 443, Taiwan; ccchen@sunrise.hk.edu.tw; 3 Department of Health and Nutrition Biotechnology, Asia University, Taichung 413, Taiwan; cychao@asia.edu.tw; 4 Department of Medical Research, China Medical University Hospital, China Medical University, Taichung 404, Taiwan; 5 Department of Forestry, National Chung-Hsing University, Taichung 402, Taiwan; taiwanfir@dragon.nchu.edu.tw; 6 School of Pharmacy, College of Pharmacy, Kaohsiung Medical University, Kaohsiung 807, Taiwan; hschang@kmu.edu.tw; 7 Graduate Institute of Natural Products, College of Pharmacy, Kaohsiung Medical University, Kaohsiung 807, Taiwan; 8 Graduate Institute of Marine Biotechnology, Department of Life Science and Institute of Biotechnology, National Dong Hwa University, Pingtung 944, Taiwan; pjsung@nmmba.gov.tw; 9 School of Chinese Pharmaceutical Sciences and Chinese Medicine Resources, China Medical University, Taichung 404, Taiwan; gjhuang@mail.cmu.edu.tw; 10 Department of Chemistry, National Taiwan University, Taipei 106, Taiwan; r93223083@ntu.edu.tw; 11 Department of Biotechnology, Asia University, Taichung 413, Taiwan

**Keywords:** Chinese herb, Taxodiaceae, *Cunninghamia konishii*, eudesmane, cadinane, sesquiterpenoid

## Abstract

Three new sesquiterpenoids, 2α-hydroxy-3,3,6α,9β-tetramethyltricyclo[4,3,2^1,4^]undecane (**1**), 11-acetoxyeudesman-4β-ol (**4**), and 2α,3β-dihydroxy-4β-methyl-6,8,10-cadinatriene (**6**), four known sesquiterpenoids (**2**, **3**, **5**, and **7**), together with eight known diterpenoids (**8**–**15**), were isolated from the wood of *Cunninghamia konishii.* Their structures were determined by detailed analysis of spectroscopic data and comparison with the data of known analogues. Four sesquiterpenoids (**1**, **4**, **5**, and **6**) and all the diterpenoids (**8**–**15**) were evaluated for inhibition of nitric oxide production in lipopolysaccharides (LPS)-activated RAW 264.7 macrophages and the results showed that compounds **10** and **15** exhibited moderate inhibitory activities against nitric oxide production.

## 1. Introduction

**Figure 1:**
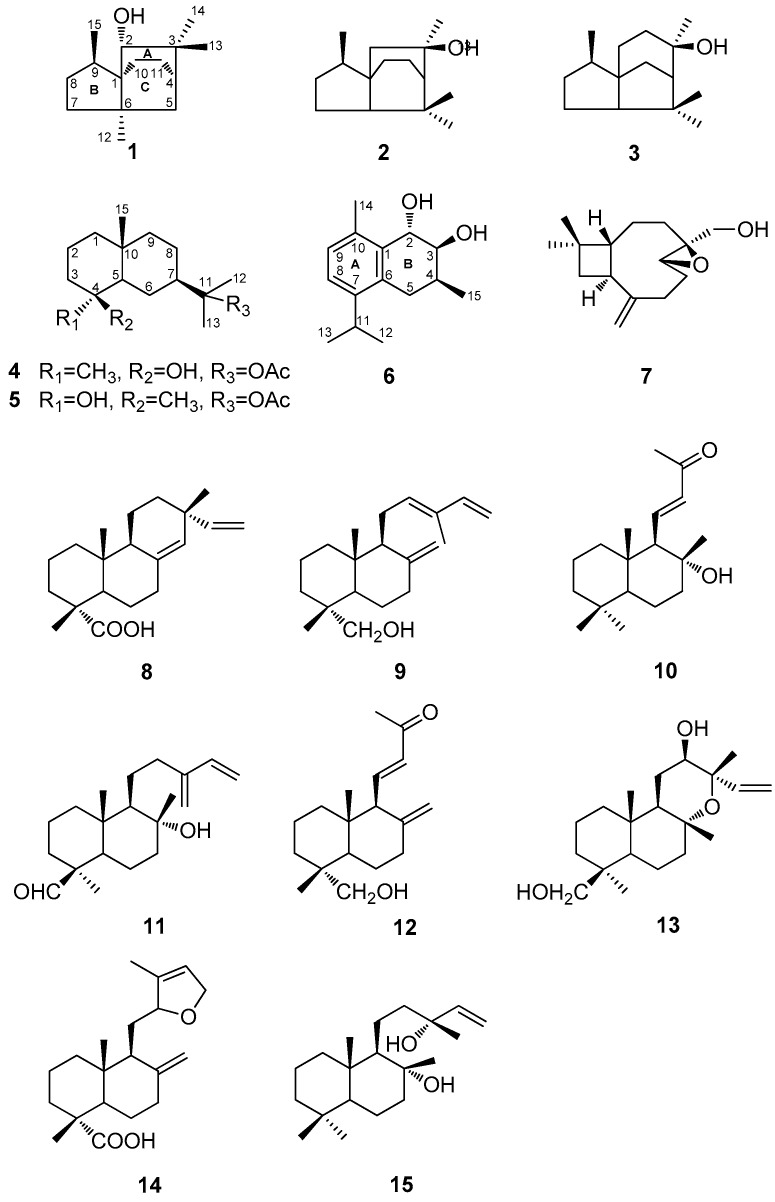
The chemical structures of compounds **1**–**15** isolated from *Cunninghamia konishii*.

The genus *Cunninghamia* contains two species occurring in eastern Asia, *Cunninghamia konishii* and *C. lanceolata*. *C. konishii*, an endemic Taiwanese coniferous tree up to 50 m tall and with a 1–2.5 m trunk diameter, grows in the northern and central forests of Taiwan at elevations ranging from 1300 to 2700 m [1]. Its wood exhibits soft, lightweight, aromatic, and rot-resistant properties, and thus is one of the best building materials and wood products available in Taiwan. A series of monoterpenes, sesquiterpenes, diterpenes, and lignans were found in its wood [2,3,4,5,6,7,8,9,10,11,12], bark [13], leaf [8], and whole plant [14], some of which have been proven to possess anti-inflammatory [10], antifungal [8,9], and cytotoxic [14] activities. As part of our program to search for secondary metabolites from this plant, we had reported the isolation and structure elucidation of 27 diterpenoids and two lignans from the wood of this plant [6,7,10,11,12,15,16]. In our continuing study of new chemicals from the wood of *C.* *konishii*, three new sesquiterpenoids, 2α-hydroxy-3,3,6α,9β-tetramethyltricyclo[4,3,2^1,4^]undecane (**1**), 11-acetoxyeudesman-4β-ol (**4**), and 2α,3β-dihydroxy-4β-methyl-6,8,10-cadinatriene (**6**), four known sesquiterpenoids (**2**, **3**, **5**, and **7**), together with three known diterpenoids (**8**–**10**), were isolated (Figure 1). Herein, we reported the extraction, isolation, and structure elucidation of compounds **1**, **4**, and **6**. Among these isolated compounds, four sesquiterpenoids (**1**, **4**, **5**, and **6**) and eight diterpenoids (**8**–**15**), including five diterpenoids that we reported previously (**11**–**15**), were evaluated for their inhibitory effects on lipopolysaccharides (LPS)-induced nitric oxide production in RAW 264.7 cells.

## 2. Results and Discussion

### 2.1. Isolation and Structural Elucidation

The MeOH extract of the wood of *C. konishii* was concentrated to give a brown residue, which was suspended in water and partitioned with EtOAc and *n*-BuOH, successively. The combined EtOAc soluble fraction was purified by repeated silica gel column chromatography and normal phase semipreparative high-performance liquid chromatography (HPLC) to obtain three new sesquiterpenoids, 2α-hydroxy-3,3,6α,9β-tetramethyltricyclo[4,3,2^1,4^]undecane (**1**), 11-acetoxyeudesman-4β-ol (**4**), and 2α,3β-dihydroxy-4β-methyl-6,8,10-cadinatriene (**6**), four known sesquiterpenoids, 3,7,7,9-tetramethyloctahydro-3a,6-ethanoinden-9-ol (**2**) [17], cedrol (**3**) [18], 11-acetoxyeudesman-4α-ol (**5**) [19], and 4β,5β-epoxy-14-hydroxy-9-*epi*-β-caryophyllene (**7**) [20], in addition to three known compounds, sandaracopimaric acid (**8**) [21], elliotinol (**9**) [22], and 8α-hydroxy-15,16-bisnorlabda-11-en-13-one (**10**) [23] (Figure 1). The identification of the known compounds was established through direct comparison with the published physical and spectral data (IR (infrared), UV (ultraviolet), MS (mass spectrum), and NMR (nuclear magnetic resonance)).

**Figure 2:**
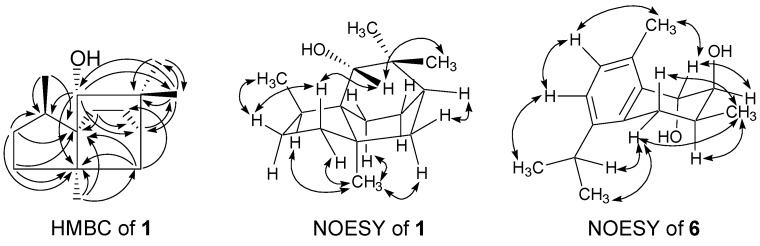
Significant HMBC (one-headed arrows) and NOESY (two-headed arrows) correlations of compounds **1** and **6**.

Compound **1** was isolated as a light yellow oil. A high resolution electron impact mass spectrometry (HR-EI-MS) molecular [M]^+^ ion at *m*/*z* 222.1980 ([M]^+^, calcd 222.1985) indicated the molecular formula of **1** to be C_15_H_26_O, showing three degrees of unsaturation. The IR spectrum demonstrated the presence of hydroxyl (3432 cm^−1^) functionality. Fifteen carbon signals were observed in the ^13^C-NMR spectrum of **1** (Table 1) and were assigned by the distortionless enhancement by polarization transfer (DEPT) experiments as four aliphatic methyl, five aliphatic methylene, two aliphatic methine, three aliphatic quaternary, and one oxygenated methine carbons. Its ^1^H-NMR spectrum (Table 1) revealed signals of the presence of one oxygenated methine (δ_H_ 3.04 (s)), three singlet methyls (δ_H_ 0.98 (s), 0.99 (s), and 1.12 (s)), and one characteristic Me-15 doublet methyl of cedrane sesquiterpenoid (δ_H_ 0.85 (d, 7.2)) [24]. From the above evidence, compound **1** was tentatively assigned as a functionalized tricycloundecane framework such as the cedrane derivative. The heteronuclear multiple bond coherence (HMBC) correlations between Me-15 (δ_H_ 0.85)/C-1 (δ_C_ 54.1 (s)) and C-9 (δ_C_ 40.7 (d)); Me-12 (δ_H_ 1.12)/C-1 and C-6 (δ_C_ 47.0 (s)); and H-8 (δ_H_ 1.17)/C-1, C-6, and C-9 help to confirm that ring B was a five-membered ring. Me-15 attached on C-9, and C-1 served as the bridgehead carbon of rings A, B, and C. The HMBC correlations between H-2 (δ_H_ 3.04)/C-4 (δ_C_ 49.7 (d)), C-6, C-9 and C-13 (δ_C_ 24.6 (q)); Me-12/C-1, C-5 (δ_C_ 34.6 (t)), C-6, and C-7 (δ_C_ 22.9 (t)); Me-13 (δ_H_ 0.99)/C-2 (δ_C_ 84.2 (d)), C-3 (δ_C_ 37.3 (s)), C-4, and C-14 (δ_C_ 29.9 (q)) suggested that ring A was a six-membered ring. The hydroxyl group located at C-2; Me-12 attached on C-6; two germinal methyls, Me-13 and 14, attached on C-3; and C-4 served as the bridgehead carbon of rings A and C. The remaining two carbon signals, together with the HMBC correlations H-10 (δ_H_ 1.51)/C-1 and C-4 and H-5 (δ_H_ 1.36)/C-11 (δ_C_ 31.2 (t)), hinted that ring C was also a six-membered ring (Figure 2). The nuclear Overhauser enhancement spectroscopy (NOESY) correlation between H-2/H_β_-7 (δ_H_ 1.13) and Me-13 indicated that the hydroxyl group at C-2 was in α orientation. The significant NOESY correlations between Me-12/H_α_-7 (δ_H_ 1.58) and Hα-10 (δ_H_ 1.51) and H_β_-8 (δ_H_ 1.75)/H_β_-7 and Me-15 hinted that Me-12 and Me-15 were in α and β orientation, respectively. Therefore, compound **1** was determined as 2α-hydroxy-3,3,6α,9β-tetramethyltricyclo[4,3,2^1,4^]undecane with a new sesquiterpene skeleton. Complete ^1^H- and ^13^C-NMR chemical shifts were established by ^1^H-^1^H correlated spectroscopy (^1^H-^1^H COSY), heteronuclear multiple-quantum coherence (HMQC), HMBC, and NOESY spectra (see Figures S3–S6 for more details).

Compound **4** was also obtained as a yellow oil, and the high resolution electron impact mass spectrometry (HR-EI-MS) data determined the molecular formula to be C_17_H_30_O_3_ (*m*/*z* 282.2195 ([M]^+^, calcd 282.2184)), indicating three degrees of unsaturation. The IR spectrum displayed the presence of carbonyl (1731 cm^−1^) and hydroxyl (3432 cm^−1^) functionalities. The ^1^H- and ^13^C-NMR spectra of **4** (Table 2) revealed resonances for an acetyl group (δ_H_ 1.95 (s); δ_C_ 22.5 (q) and 170.5 (s)), and four singlet methyls (δ_H_ 1.00 (s), 1.14 (s), 1.42 (s), and 1.43 (s)). Seventeen carbon signals including two carbon signals of the acetyl group were observed in the ^13^C-NMR spectrum of **4** and were assigned by DEPT experiments as four alphatic methyl, six alphatic methylene, two alphatic methine, one aliphatic quaternary, two quaternary oxygenated, one ester carbonyl, and one acetylic methyl carbons. Take out one degree of unsaturation contributed from the carbonyl group, and the remaining two degrees of unsaturation, along with the information of the 15 carbon skeleton, hinted **4** would be a sesquiterpenoid derivative with a bicyclic structure. Compound **4** was thus tentatively proposed to be a eudesmane sesquiterpenoid. Comparison of the ^1^H- and ^13^C-NMR data with those of the known compound, 4-epicryptomeridiol [25], indicated that both compounds exhibited identical structure in the eudesmane skeleton, with the only difference occurring in the signals of an acetoxy group at C-11 in **4** instead of that of a hydroxyl group in 4-epicryptomeridiol (**4a**). After the 10% KOH alkaline hydrolysis, **4** could be transferred to 4-epicryptomeridiol. Compound **4** was accordingly determined to be 11-acetoxyeudesman-4β-ol.

Compound **6** was obtained as a light yellow oil. The IR spectrum of **6** showed bands that were attributable to hydroxyl (3416 cm^−1^) and aromatic (1640 and 1460 cm^−1^) functionalities. The HR-EI-MS of **6** showed a molecular ion at *m/z* 234.1622, which corresponded to the molecular formula C_15_H_22_O_2_, indicating five degrees of unsaturation. The ^1^H- and ^13^C-NMR spectra of **6** (Table 2) revealed resonances for an isopropyl group [δ_H_ 1.19 (3H, d, *J* = 6.8 Hz), 1.20 (3H, d, *J* = 6.8 Hz), 3.15 (1H, sept, *J* = 6.8 Hz); δ_C_ 23.1 (q), 23.7 (q), 28.0 (d)], a benzylic methylene [δ_H_ 2.45 (1H, dd, *J* = 17.2 and 10.4 Hz), 2.80 (1H, d, *J* = 17.2 and 6.0 Hz); δ_C_ 28.7 (t)] and a benzylic methyl [δ_H_ 2.37 (3H, s); δ_C_ 18.7 (q)], two *ortho*-coupled aromatic protons [δ_H_ 7.07 (1H, d, *J* = 8.0 Hz), 7.15 (1H, d, *J* = 8.0 Hz); δ_C_ 124.8 (d), 128.9 (d)], and two oxymethines [δ_H_ 3.93 (1H, dd, *J* = 3.6 and 2.0 Hz), 4.79 (1H, d, *J* = 3.6 Hz); δ_C_ 73.6 (d), 69.8 (d)]. According to the above spectral characteristics, compound **6** exhibited a bicyclic sesquiterpenoid with a tetrasubstituted benzene ring, and was thus tentatively proposed to be a cadinatriene derivative. The position of the substituents in ring A and the relative configurations of the sterogenic C-atoms in ring B were determined by significant NOE correlations between H_α_-5 (δ_H_ 2.80)/H-11 (δ_H_ 3.15) and Me-15 (δ_H_ 1.17); Me-12 (δ_H_ 1.20)/H-8 (δ_H_ 7.15); Me-14 (δ_H_ 2.37)/H-2 (δ_H_ 4.79) and H-9 (δ_H_ 7.07); and Me-15/H_β_-5 (δ_H_ 2.45) in the NOESY spectrum (Figure 2). The smaller coupling constants of two oxymethines [δ_H_ 3.93 (1H, dd, *J* = 3.6 and 2.0 Hz), 4.79 (1H, d, *J* = 3.6 Hz)] hinted that the two neighboring hydroxyl groups in ring B were all in axial orientation. The spectral data of **6** was in good agreement with those reported for the known compound konishiol [14], except for an incorrect published ^1^H-NMR data of Hα-5 (δ_H_ 2.45, instead of δ_H_ 2.80 in the literature). The specific optical rotation of **6** was +9.7, compared to −8.9 of konishiol, and **6** was thus determined as the enantiomer of konishiol, 2α,3β-dihydroxy-4β-methyl-6,8,10-cadinatriene, namely as *ent*-konishiol.

### 2.2. Inhibitory Activity against Nitric Oxide Production

Nitric oxide (NO) is derived from the oxidation of l-arginine by NO synthase (NOS) and is recognized as a mediator and regulator in biological actions, especially in inflammatory responses [26]. In inflammation and carcinogenesis conditions, there is an increased production of NO by inducible NO synthase (iNOS) [27]. Thus, inhibitors of NO might be of therapeutic importance in preventing pathological conditions catalyzed by inflammation. Macrophages contain various chemical mediators that may be responsible for several inflammatory stages and have been expected to be an origin of inflammation [28]. iNOS mainly exists in macrophages and can be induced by pro-inflammatory agents lipopolysaccharides (LPS). LPS can significantly increase the level of nitric oxide (NO) in macrophages through activation of iNOS [29]. In this study, the inhibitory activity toward NO production of four sesquiterpenoids (**1**, **4**, **5**, and **6**) and eight diterpenoids (**8**–**15**) was evaluated by measurement of nitrite/nitrate in LPS-stimulated RAW 264.7 cells. To search for the appropriate concentrations for the above assay, these 12 compounds were first tested their cytotoxic activity against the RAW 264.7 cells, and no significant cytotoxic activities were observed under all tested concentrations (Table 3). Furthermore, compounds **10** and **15** exhibited moderate inhibitory effects on lipopolysaccharides (LPS)-induced nitric oxide production in RAW264.7 cells with IC_50_ values of 11.44 and 13.07 μg/mL, respectively (Table 3). Indomethacin is related to the inhibition of the cyclooxgenase 2 enzyme which synthesizes prostaglandin and was determined as a positive control (IC_50_ value of 65.4 μg/mL).

## 3. Experimental Section

### 3.1. Chemicals

LPS (endotoxin from *Escherichia coli*, serotype 0127:B8), indomethacin, MTT(3-[4,5-dimethylthiazol-2-yl]-2,5-diphenyltetrazolium bromide) and other chemicals were purchased from Sigma Chemical Co. (St. Louis, MO, USA).

### 3.2. General

The UV spectra were obtained on a Shimadzu UV-1601PC spectrophotometer (Shimadzu Corp., Kyoto, Japan). Optical rotations were measured with a JASCO DIP-180 digital spectropolarimeter (JASCO Inc., Tokyo, Japan). The IR spectra were recorded on a Nicolet 510P FT-IR spectrometer (Thermo Scientific Inc., Waltham, MA, USA). The 1D- and 2D-NMR spectra were measured with a Varian-Unity-Plus-400 spectrometer (Varian Inc., Palo Alto, CA, USA). Chemical shift values are given in ppm with reference to solvent (TMS as standard) and coupling constants (*J*) are given in Hz. The 2D-NMR spectra were recorded by using standard pulse sequences. EI-MS and HR-EI-MS were recorded on a JEOL SX-102A mass spectrometer (JEOL Ltd., Tokyo, Japan). Column chromatography was carried out on Merck Si gel (230–400 mesh ASTM, Merck, Darmstadt, Germany). TLC (thin-layer chromatography) analysis was carried out using aluminum pre-coated Si plates (Silica Gel 60 F-254; Merck) and the spots were detected by spraying with 5% H_2_SO_4_ and then heating at 100 °C. Semi-preparative HPLC was performed using a normal phase column (LiChrosorb Si 60, 7 μm, 250 × 10 mm; Merck & Co., Inc.) on a LDC Analytical-III system.

### 3.3. Plant Material

The wood of *C. konishii* was collected at Luantashan, Nantau County, Taiwan, in December 1996. The material was identified by Prof. Shao-Shun Ying, Department of Forestry, National Taiwan University. A voucher specimen (013492) has been deposited at the Herbarium of the Department of Botany, National Taiwan University, Taipei, Taiwan.

### 3.4. Extraction and Isolation

Dried wood (6.5 kg) of *C. konishii* was crushed into pieces and extracted by immersing in MeOH (60 L × 3) at r.t. for seven days each time. The combined MeOH extract was evaporated under reduced pressure at 45 °C to afford a brown crude viscous residue (60.2 g), which was suspended in H_2_O (500 mL), and then partitioned sequentially, using hexane (500 mL × 3), EtOAc (500 mL × 4), and BuOH (500 mL × 3) as solvent. The EtOAc fraction (15.6 g) was chromatographed on silica gel (450 g; 4.5 × 60 cm) using *n*-hexane–EtOAc (10:0, 9:1, 4:1, 7:3, 3:2, 1:1, 2:3, 3:7, 1:4, and 0:10) and EtOAc–MeOH (5:1) mixtures as solvent systems to obtain 11 fractions. Fr. 2 (150 mg) from *n*-hexane–EtOAc (9:1) elution was identified as a mixture of **1**–**3**. Further purification by semi-preparative HPLC (hexane/CH_2_Cl_2_/EtOAc 10:5:1) gave **1** (2.2 mg), **2** (1.8 mg), and **3** (3.2 mg). Fr. 4 (320 mg) from *n*-hexane–EtOAc (7:3) elution was identified as a crude **9**. Further purification by semi-preparative HPLC (hexane/CH_2_Cl_2_/EtOAc/*i*PrOH 8:2:1:0.2) gave **9** (1.2 mg). Fr. 6 (270 mg) from *n*-hexane–EtOAc (1:1) elution was identified as a mixture of **4**, **5**, **7**, and **8**. Further purification by semi-preparative HPLC (hexane/CH_2_Cl_2_/EtOAc/*i*PrOH 10:5:1:0.2) gave **4** (3.1 mg), **5** (2.2 mg), **7** (1.3 mg), and **8** (2.0 mg). Fr. **8** (310 mg) from *n*-hexane–EtOAc (3:7) elution was identified as a mixture of **6** and **10**. Further purification by semi-preparative HPLC (hexane/EtOAc/*i*PrOH 3:1:0.3) gave **6** (2.1 mg) and **10** (1.6 mg).

*2α-Hydroxy-3,3,6α,9β-tetramethyltricyclo[4,3,2^1,4^]undecane* (**1**). Light yellow oil; ${\left[\alpha \right]}_{D}^{25}$ = −38.7 (*c* = 0.20, CHCl_3_); EI-MS (70 eV) *m*/*z* (rel. int.%): 222 ([M]^+^, 4), 204 ([M − H_2_O]^+^, 38), 203 (68), 189 (98), 183 (57), 161 (100); HR-EI-MS *m*/*z*: 222.1980 [M]^+^ (calcd for C_15_H_26_O, 222.1985); IR (KBr) *ν*max: 3432, 1460, 1376, 1015 cm^−1^; ^1^H-NMR and ^13^C-NMR (400/100 MHz, in CDCl_3_): see Table 1.

*11-Acetoxyeudesman-4β-ol* (**4**). Light yellow oil; ${\left[\alpha \right]}_{D}^{25}$ = +3.8 (*c* = 0.21, CHCl_3_); EI-MS (70 eV) *m*/*z* (rel. int.%): 282 ([M]^+^, 3), 259 ([M − CH_3_COOH]^+^, 25), 207 (28), 204 (100), 189 (24); HR-EI-MS *m/z*: 282.2195 [M]^+^ (calcd for C_17_H_30_O_3_, 282.2184); IR (KBr) ν_max_: 3432, 1731, 1454, 1370, 1260, 1125, 1015 cm^−1^; ^1^H-NMR and ^13^C-NMR (400/100 MHz, in CDCl3): see Table 2.

*2α,3β-Dihydroxy-4β-methyl-6,8,10-cadinatriene* (**6**). Light yellow oil; ${\left[\alpha \right]}_{D}^{26}$ = +9.7 (*c* = 0.19, CHCl_3_); EI-MS (70 eV) *m*/*z* (rel. int.%): 234 ([M]^+^, 64), 216 (44), 201 (43), 187 (42), 173 (41), 161 (62); HR-EI-MS *m*/*z*: 234.1622 [M]^+^ (calcd for C_15_H_22_O_2_, 234.1621); UV_max_ (CH_3_OH): 204, 261 nm; IR (KBr) ν_max_: 3416, 1640, 1467, 1387, 1049, 997 cm^−1^; ^1^H-NMR and ^13^C-NMR (400/100 MHz, in CDCl_3_): see Table 2.

### 3.5. Cell Culture

A murine macrophage cell line RAW264.7 (BCRC No. 60001) was obtained from the Bioresources Collection and Research Center (BCRC) of the Food Industry Research and Development Institute (Hsinchu, Taiwan). Cells were maintained in Dulbecco’s Modified Eagle Medium (DMEM, Sigma, St. Louis, MO, USA) supplemented with 10% fetal bovine serum (FBS, Sigma). Cells were cultured in a humidified atmosphere with 5% CO_2_ at 37 °C and subcultured every three days at a dilution of 1:5 using 0.05% trypsin-0.02% EDTA in Ca^2+^-, Mg^2+^-free phosphate-buffered saline (DPBS).

### 3.6. Measurement of Nitric Oxide/Nitrite

The anti-inflammatory activity of compounds was evaluated by using a nitric oxide (NO) inhibitory activity assay. As a stable NO metabolite, production of NO was indirectly determined by Griess reaction to measure the concentration of nitrite in the culture medium. The cells were incubated with test compounds (0, 2.5, 5, 10, 20 and 40 μg/mL) in the presence of LPS (100 ng/mL) at 37 °C for 24 h. Then, cells were dispensed into 96-well plates, and 100 μL of each supernatant was reacted with an equal volume of Griess reagent (1% sulfanilamide, 0.1% naphthyl ethylenediamine dihydrochloride and 5% phosphoric acid) and incubated at room temperature for 10 min. Absorbance was then measured at 540 nm using a Micro-Reader (Molecular Devices Orleans Drive, Sunnyvale, CA, USA). A standard curve was generated, using freshly prepared 0–100 μM potassium nitrate dissolved in assay buffer, to quantitate unknown nitrite in samples.

### 3.7. Cell Viability

Cells (2 × 10^5^) were cultured in 96-well plate containing DMEM supplemented with 10% FBS. After 24 h of cells incubation, cells were cultured with test compounds in the presence of 100 ng/mL LPS (lipopolysaccharide) for 24 h. Untreated cells served as the control. After that, the cells were washed twice with DPBS and 100 μL of 0.5 mg/mL MTT was added to each well for further 2 h incubation at 37 °C. The medium was then discarded, and the colored crystals of produced formazan were dissolved in 100 μL dimethyl sulfoxide (DMSO). After 30 min incubation, the absorbance was measured at 570 nm on a microplate reader (Molecular Devices).

### 3.8. Statistical Analysis

The data is expressed as means ± standard errors (SE). The IC_50_ values were calculated from the dose curves using a non-linear regression algorithm (SigmaPlot 8.0; SPSS Inc., Chicago, IL, USA, 2002). Statistical evaluation was carried out by one-way analysis of variance (ANOVA followed by Scheffe’s multiple range tests).

## 4. Conclusions

Three new sesquiterpenoids, 2α-hydroxy-3,3,6α,9β-tetramethyltricyclo[4,3,2^1,4^]undecane (**1**), 11-acetoxyeudesman-4β-ol (**4**), and 2α,3β-dihydroxy-4β-methyl-6,8,10-cadinatriene (**6**), four known sesquiterpenoids (**2**, **3**, **5**, and **7**), together with eight known diterpenoids (**8**–**15**), were isolated from the wood of *C. konishii*. Among them, four sesquiterpenoids (**1**, **4**, **5**, and **6**) and eight diterpenoids (**8**–**15**), five of which we reported previously (**11**–**15**), were evaluated for their anti-inflammatory activity and the results showed that compounds **10** and **15** exhibited moderate inhibitory effects on lipopolysaccharides (LPS)-induced nitric oxide production in RAW264.7 cells. This investigation of secondary metabolites may contribute to a better understanding of the chemical characteristics of *C. konishii.*

As to the biological activity, these 12 compounds (**1**, **4**, **5**, **6,** and **8**–**15**) exhibited no significant cytotoxic activity at all tested concentrations. Compounds **10** and **15** showed stronger NO production inhibition than the other diterpenoids (**8**, **9**, **11**, **12**, **13**, and **14**). A hydroxyl group at C-8 served as the active site, derived from the hydration of a double bond at C-8 and C-17, and may play an important role in their inhibitory activities against NO production.

## Figures and Tables

**Table 1:** NMR (nuclear magnetic resonance) data (CDCl_3_) of compound **1**; δ in ppm, *J* in Hz.

Position	δ_H_^a^	δ_C_^b^	HMBC (^1^H→^13^C)
1		54.1	
2	3.04 (s)	84.2	C-4, C-6, C-9, C-13
3		37.3	
4	1.61 (m)	49.7	C-13, C-14
5	1.59 (m) 1.36 (dd, 14.0, 4.0)	34.6	C-4, C-6, C-11
6		47.0	
7	1.58 (m), 1.13 (m)	22.9	C-1, C-8, C-9
8	1.75 (ddd, 13.6, 6.5, 4.3), 1.17 (ddd, 13.6, 6.8, 2.0)	33.5	C-6, C-9, C-15
9	1.70 (m)	40.7	
10	1.51 (ddd, 13.2, 6.8, 4.3), 1.05 (ddd, 13.2, 6.5, 2.0)	41.0	C-1, C-4
11	1.92 (m), 1.88 (m)	31.2	
12	1.12 (s)	25.7	C-1, C-5, C-6, C-7
13	0.99 (s)	24.6	C-2, C-3, C-4, C-13
14	0.98 (s)	29.9	C-2, C-3, C-4, C-14
15	0.85 (d, 7.2)	19.8	C-1, C-9, C-10

Recorded at ^a^ 500 MHz (^1^H); ^b^ 125 MHz (^13^C).

**Table 2:** NMR (nuclear magnetic resonance) data (CDCl_3_) of compounds **4** and **6**; δ in ppm, *J* in Hz.

Position	4		6	
	δ_H_^a^	δ_C_^b^	δ_H_^a^	δ_C_^b^
1	1.65 (m), 1.03 (m)	41.5		132.4
2	1.80 (m), 1.41 (m)	18.1	4.79 (d, 3.6)	69.8
3	1.32 (m), 1.08 (m)	43.6	3.93 (dd, 3.6, 2.0)	73.6
4		72.1	2.25 (m)	27.4
5	1.03 (dd, 10.4, 5.2)	51.5	2.45 (dd, 17.2, 10.4), 2.80 (dd, 17.2, 6.0)	28.7
6	1.48 (m), 1.35 (m)	22.0		133.5
7	1.97 (m)	46.9		144.4
8	1.68 (m), 1.18 (m)	21.2	7.15 (d, 8.0)	124.8
9	1.37 (m), 1.09 (m)	41.5	7.07 (d, 8.0)	128.9
10		33.7		136.3
11		85.3	3.15 (sept, 6.8)	28.0
12	1.43 (s)	23.7	1.20 (d, 6.8)	23.7
13	1.42 (s)	23.6	1.19 (d, 6.8)	23.1
14	1.14 (s)	30.2	2.37 (s)	18.7
15	1.00 (s)	18.7	1.17 (d, 7.2)	17.8
O*C*OCH_3_		170.5		
OCO*CH*_3_	1.95 (s)	22.5		

Recorded at ^a^ 400 MHz (^1^H); and ^b^ 100 MHz (^13^C).

**Table 3:** Cell viability and *in vitro* decrease of nitric oxide production of compounds **1**, **4**, **5**, **6** and **8**–**15** in LPS-stimulated RAW 264.7 cells.

Compound	Cytotoxicity IC_50_ (μg/mL)	Inhibition of NO Production IC_50_ (μg/mL)
**1**	>20	>20
**4**	>20	>20
**5**	>20	>20
**6**	>20	>20
**8**	>20	>20
**9**	>20	>20
**10**	>20	11.44 ± 0.62
**11**	>20	>20
**12**	>20	>20
**13**	>20	>20
**14**	>20	>20
**15**	>20	13.07 ± 0.55
indomethacin	>100	65.4 ± 1.80

Values are expressed as mean ± SD of three replicates.

## Supplementary Materials

Supplementary materials can be accessed at: http://www.mdpi.com/1420-3049/21/4/490/s1.
