# Supplementary material for: Sesquiterpenoids and Diterpenoids from the Wood of Cunninghamia konishii and Their Inhibitory Activities against NO Production
Source: Molecules. 2016 Apr 13;21(4):490. doi: 10.3390/molecules21040490 (PMC6273599; doi:10.3390/molecules21040490)
Supplement: Supplementary file 1 [file molecules-21-00490-s001.pdf]

Supplementary Materials: Sesquiterpenoids and Diterpenoids from the Wood of *Cunninghamia konishii* and Their Inhibitory Activities against NO Production

Chi-I Chang, Chien-Chih Chen, Che-Yi Chao, Sheng-Yang Wang, Hsun-Shuo Chang, Ping-Jyun Sung, Guan-Jhong Huang, Yen-Cheng Li and Yueh-Hsiung Kuo

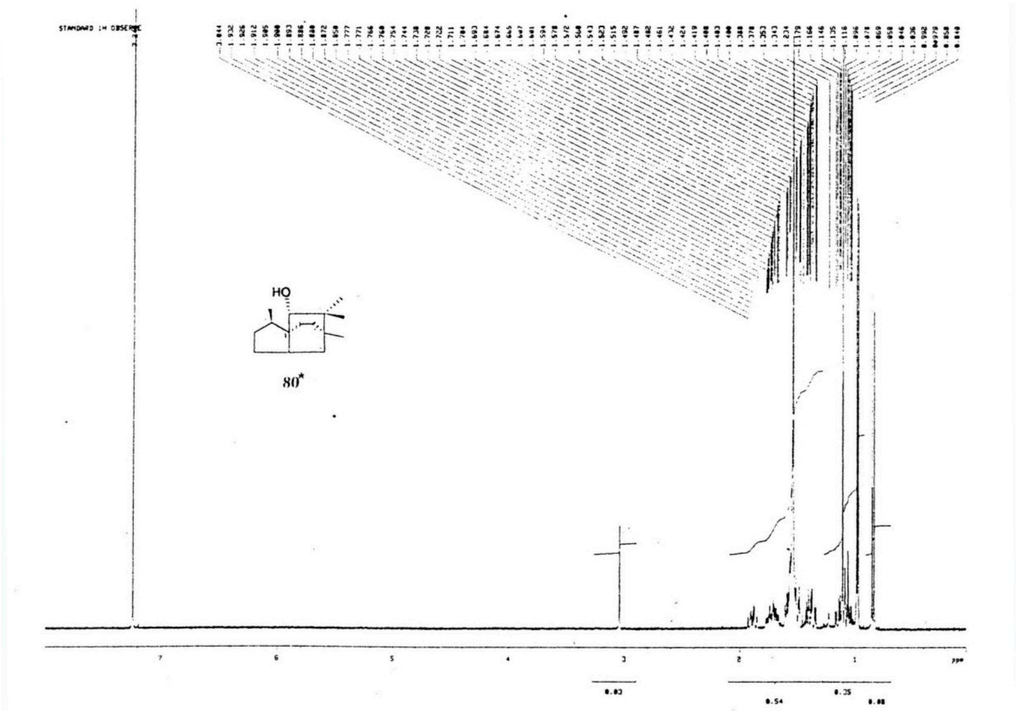

Figure S1. ^{1}H-NMR spectrum of compound **1** (400 MHz, $CDCl_3$).

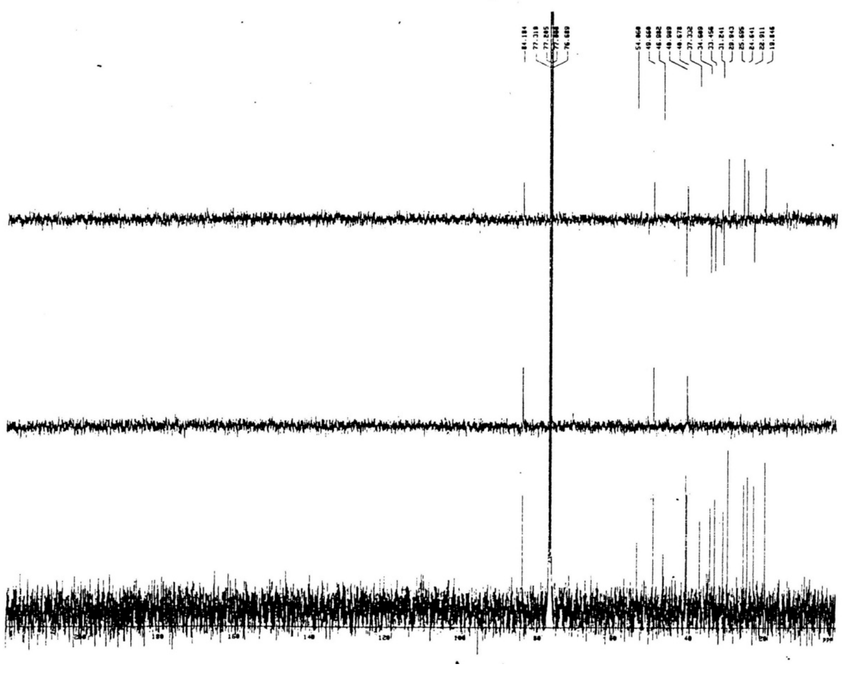

Figure S2. ^{13}C-NMR spectrum of compound **1** (100 MHz, $CDCl_3$).

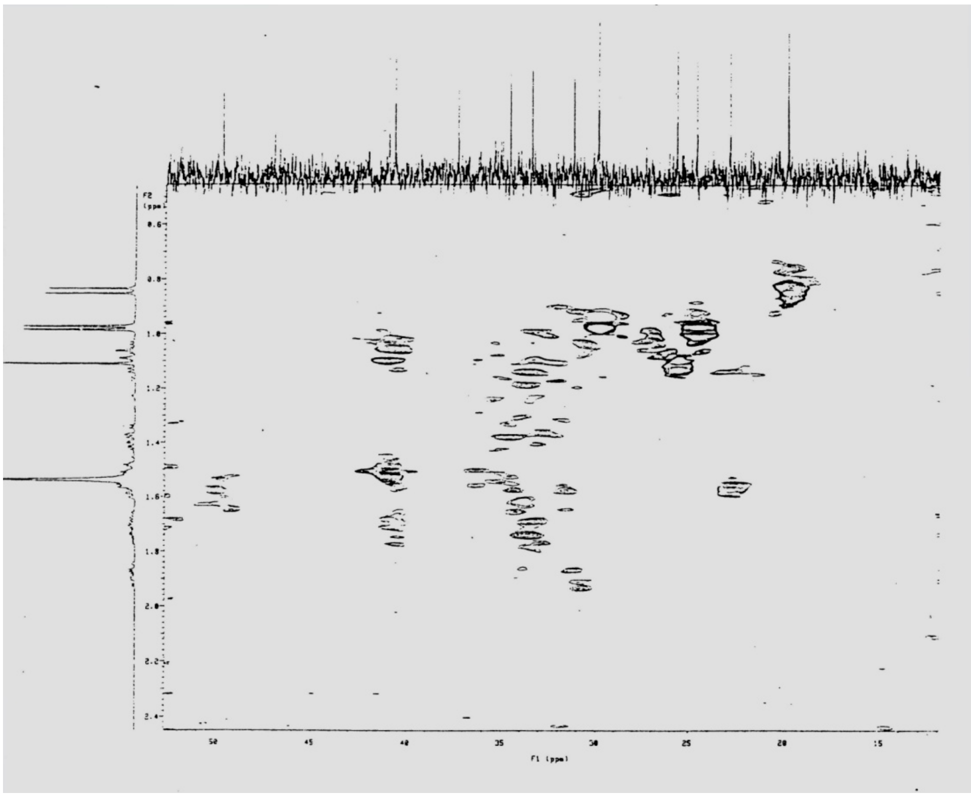

Figure S3. HMQC spectrum of compound **1**.

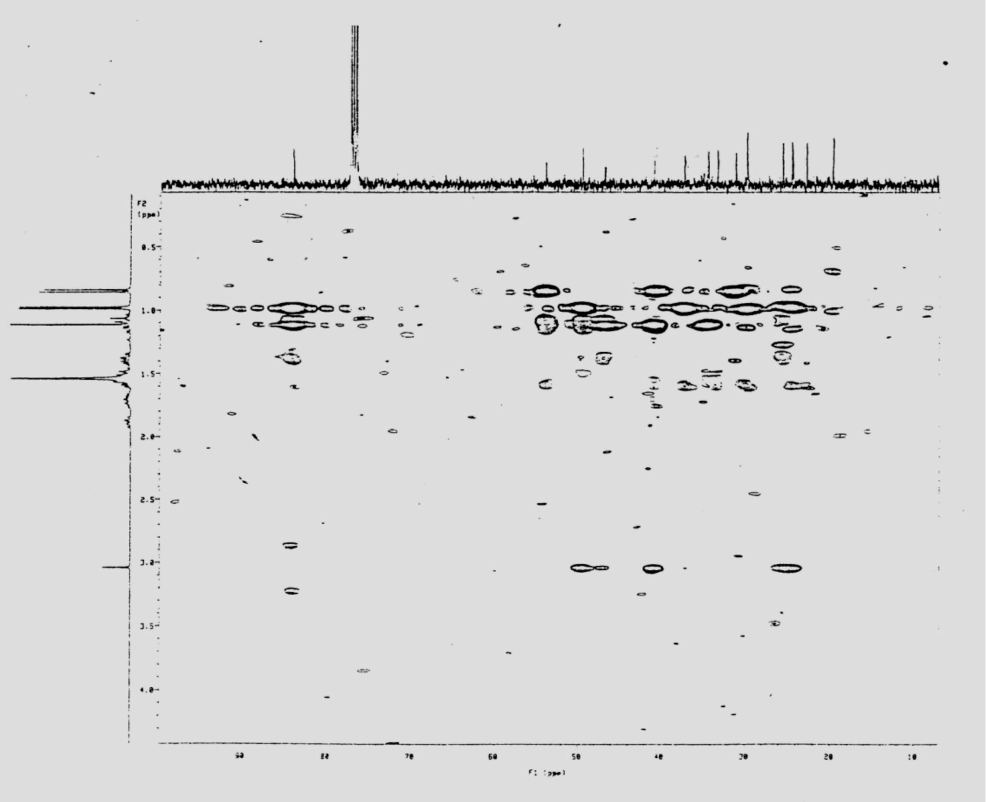

Figure S4. HMBC spectrum of compound **1**.

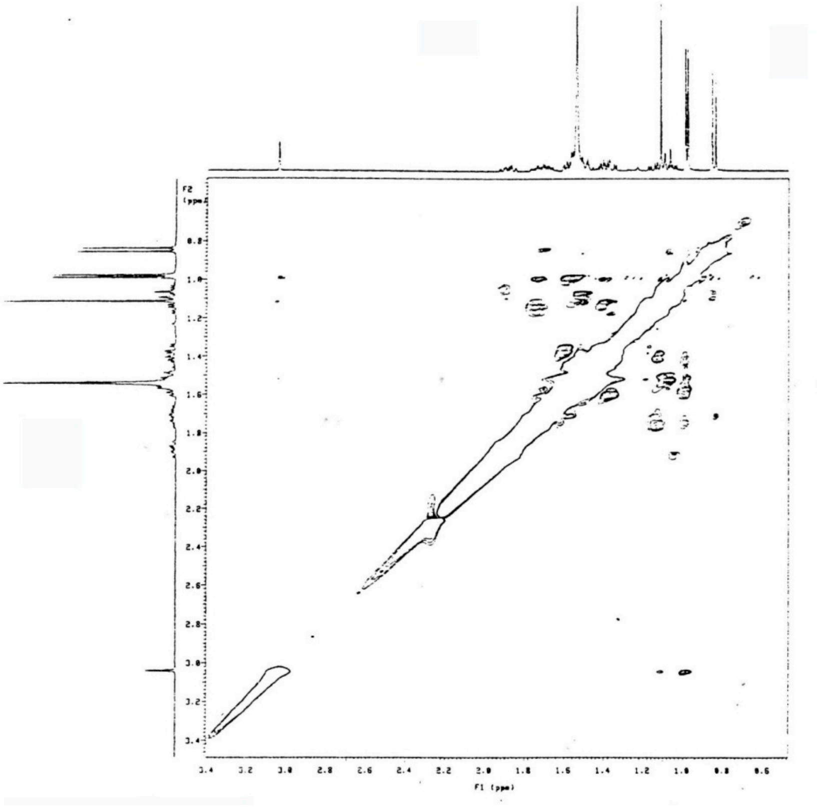

Figure S5. NOSEY spectrum of compound **1**.

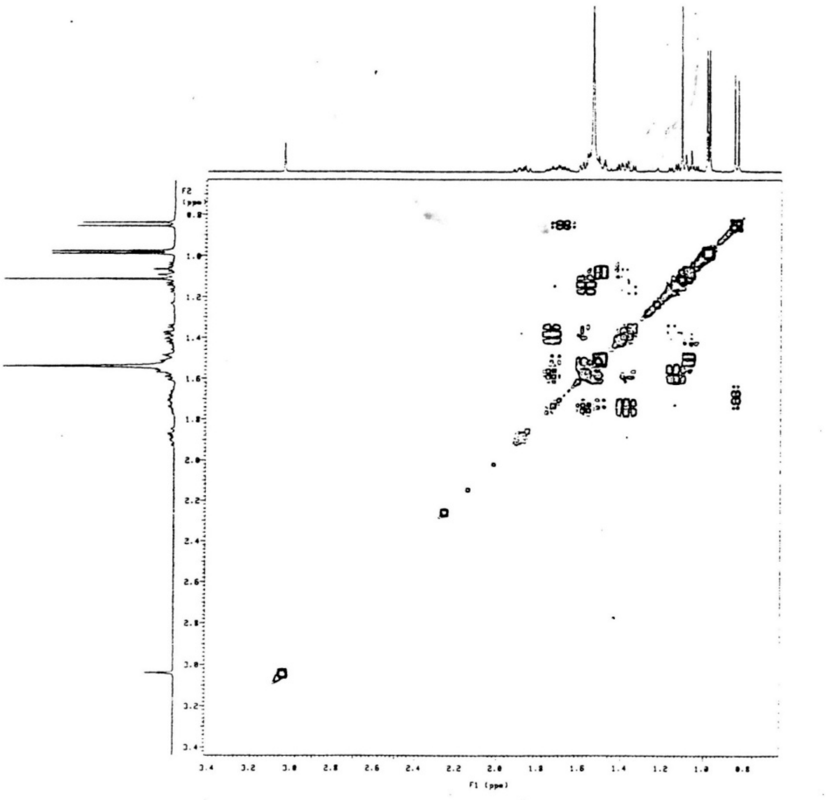

Figure S6. 1H-1H COSY spectrum of compound **1**.

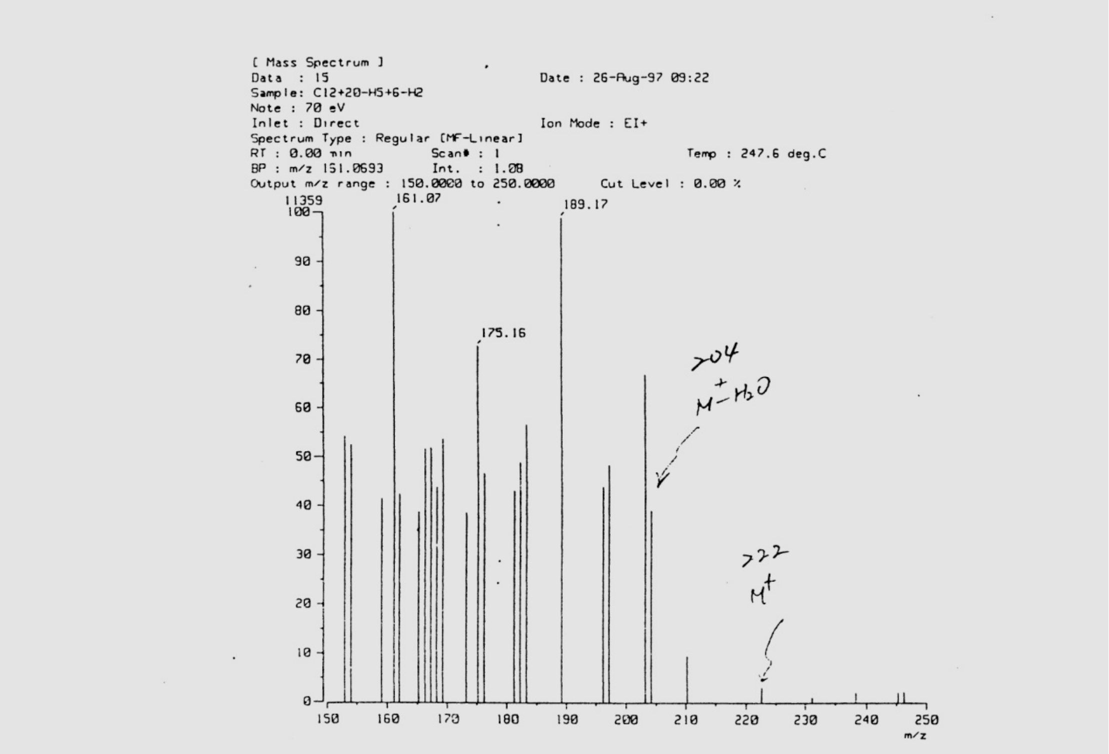

Figure S7. Mass spectrum of compound **1**.

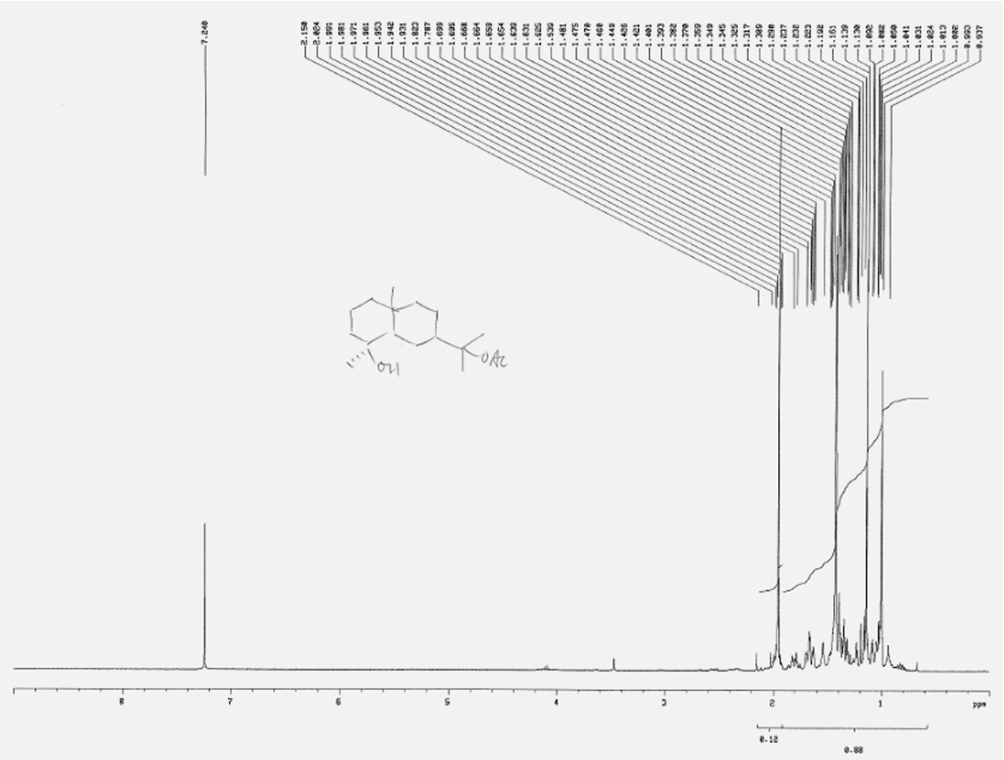

Figure S8. ^{1}H-NMR spectrum of compound **4** (400 MHz, $CDCl_3$).

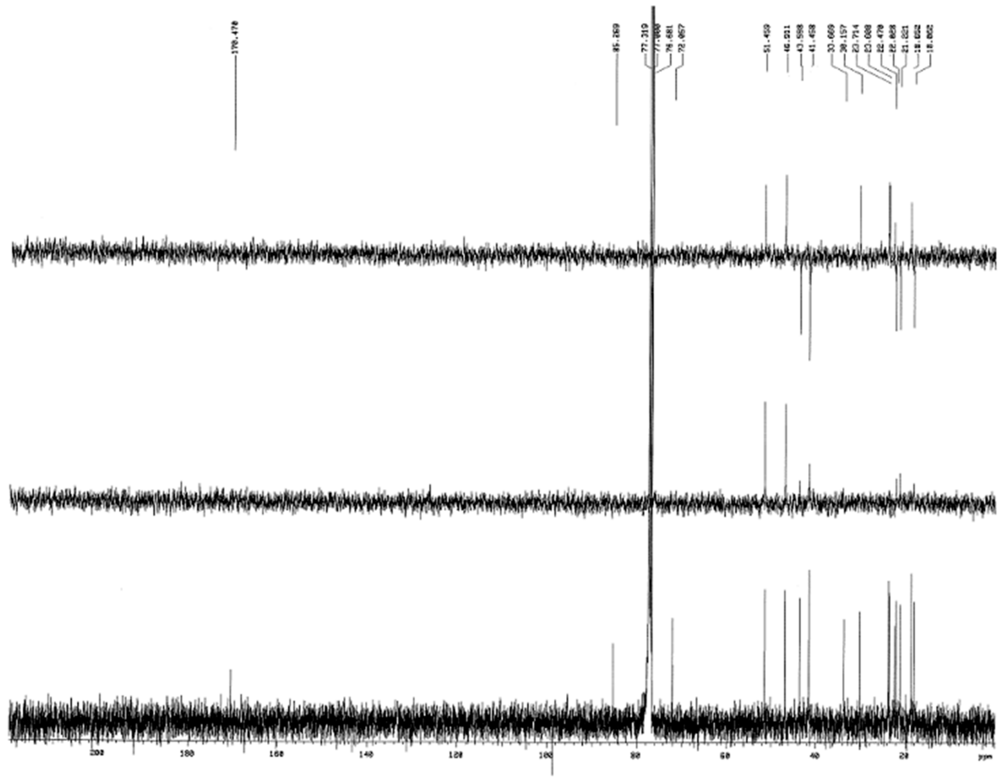

Figure S9. ^{13}C-NMR spectrum and DEPT of compound **4** (100 MHz, $CDCl_3$).

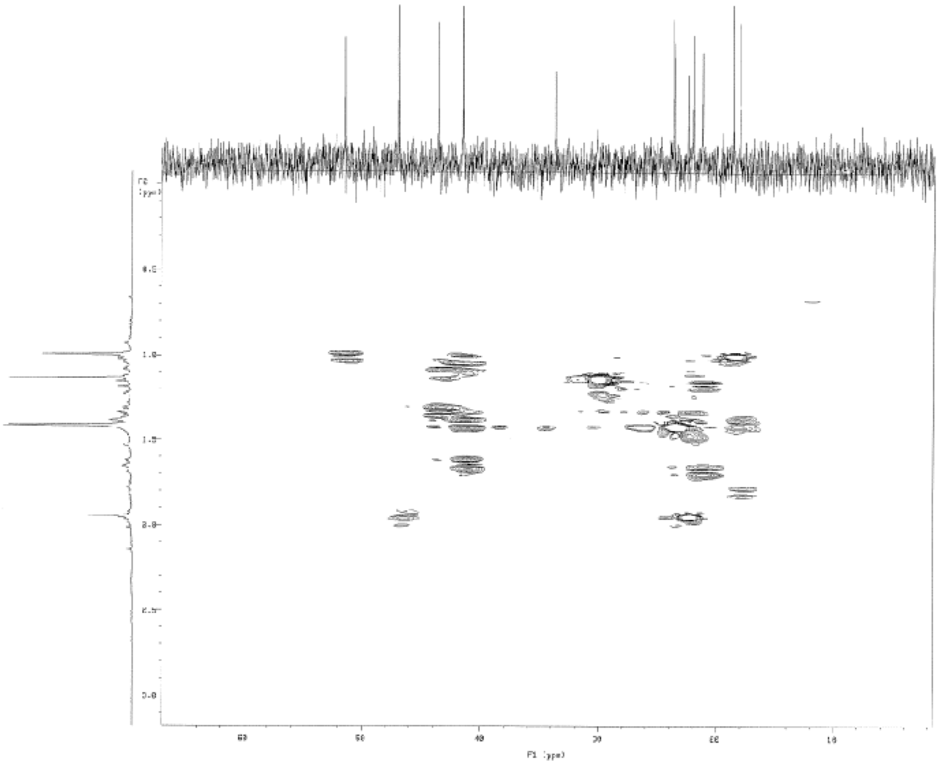

Figure S10. HMQC spectrum of compound **4**.

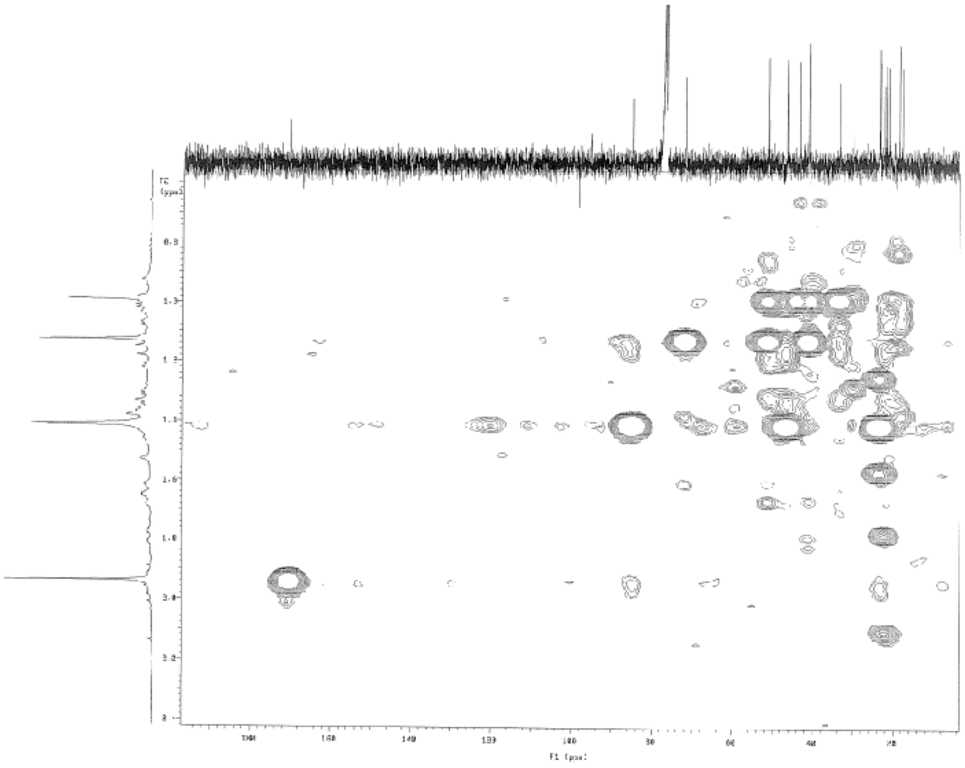

Figure S11. HMBC spectrum of compound **4**.

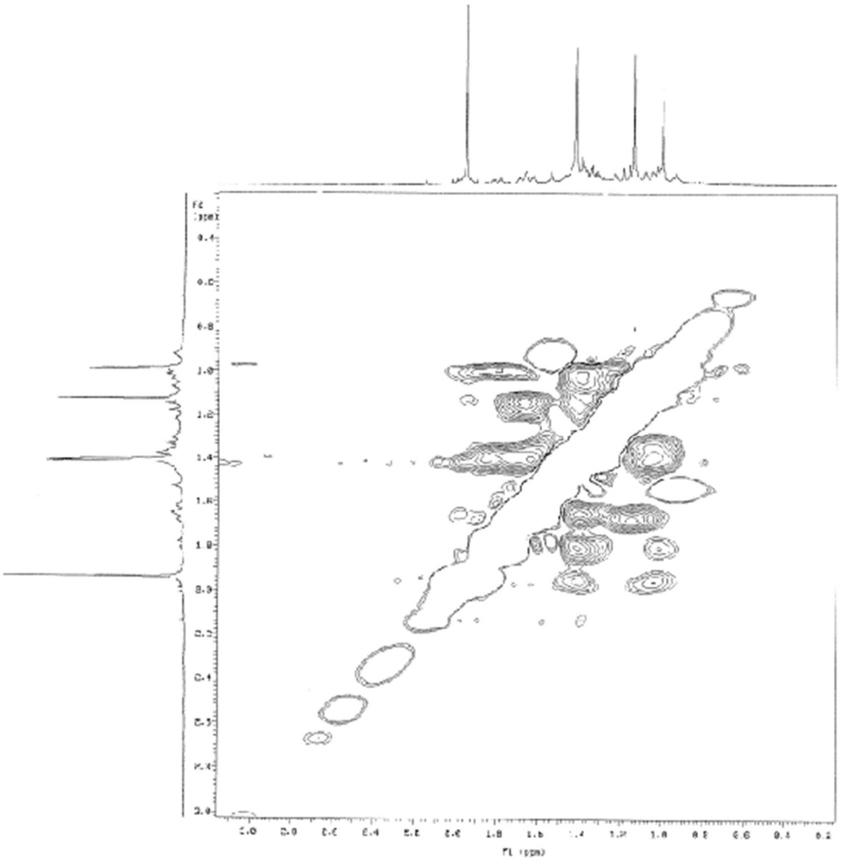

Figure S12. NOSEY spectrum of compound **4**.

```
[ Elemental Composition ]
Data   : 14                           Date : 17-Sep-97 10:29
Sample: C39+45-9-H5-H2
Note : 70 eV
Inlet : Direct                        Ion Mode : EI+
RT : 0.75 min                         Scan#: 10
Elements : C 40/0, H 50/3, O 3/0
Mass Tolerance      : 10ppm, 5mmu if m/z < 500, 20mmu if m/z > 2000
Unsaturation (U.S.) : -0.5 - 10.0

Observed m/z  Int%   Err[ppm / mmu]    U.S.  Composition
  222.1978    24.5     -2.7 /  -0.6     3.0  C 15 H 26 O

  207.1861    28.4

  204.1983   100.0

  189.1730    23.6
```

```
[ Mass Spectrum ]
Data   : 14                         Date : 17-Sep-97 10:29
Sample: C39+45-9-H5-H2
Note : 70 eV
Inlet : Direct                      Ion Mode : EI+
Spectrum Type : Regular [MF-Linear]
RT : 0.75 min          Scan# : 10                    Temp : 19.3 deg.C
BP : m/z 204.1983      Int.   : 32.95
Output m/z range : 175.0000 to 285.0000      Cut Level : 0.00 %
```

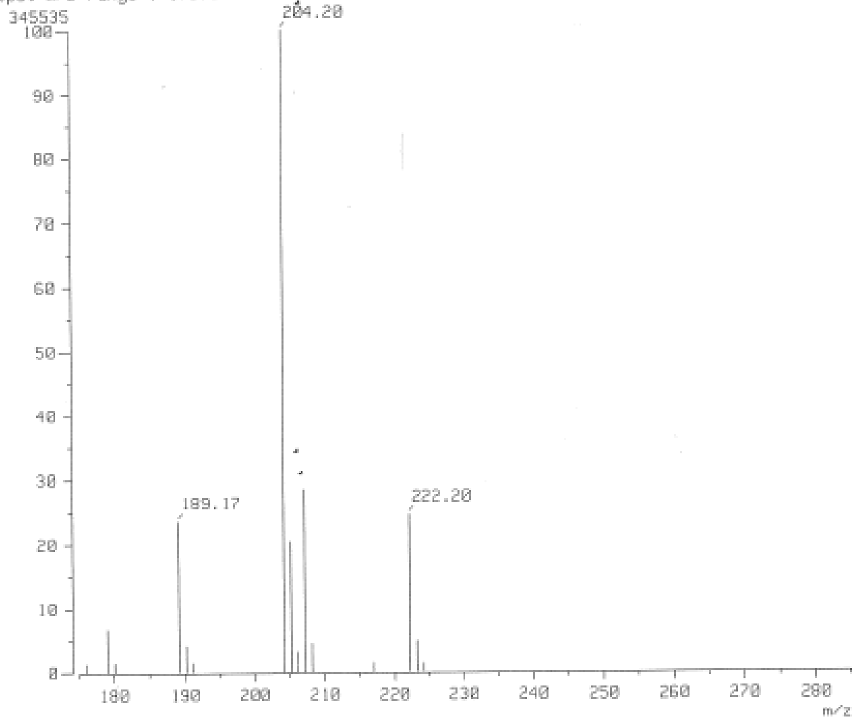

Figure S13. Mass spectrum of compound **4**.

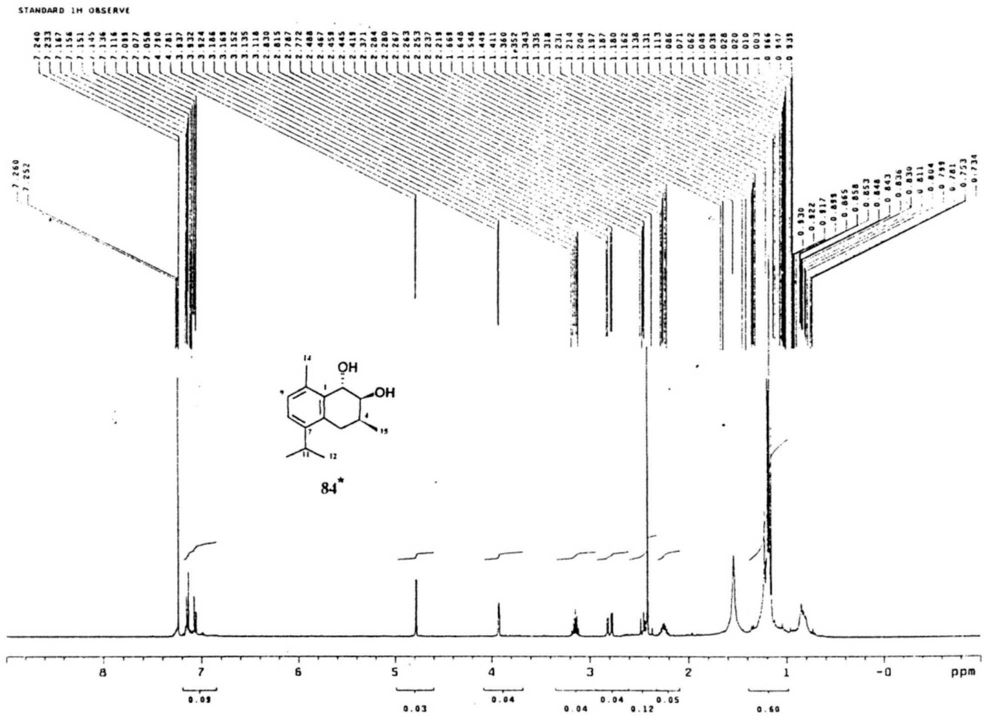

Figure S14. ^{1}H-NMR spectrum of compound **6** (400 MHz, $CDCl_3$).

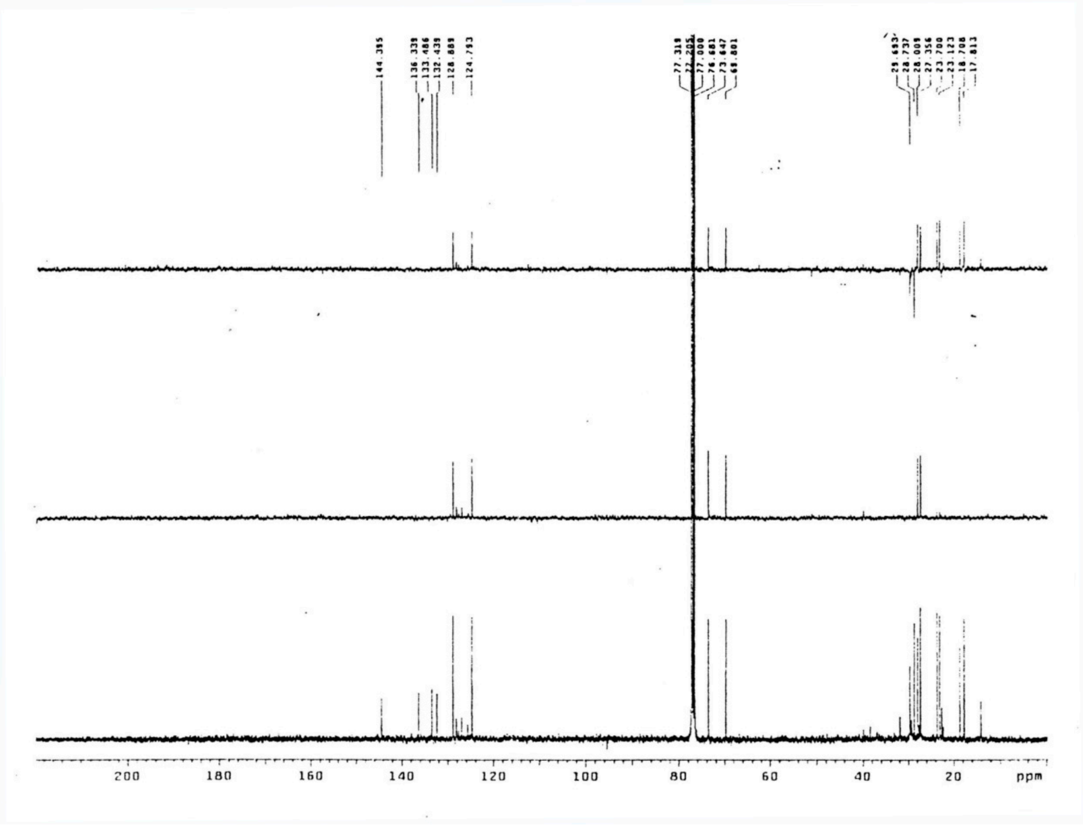

Figure S15. ^{13}C-NMR spectrum and DEPT of compound **6** (100 MHz, $CDCl_3$).

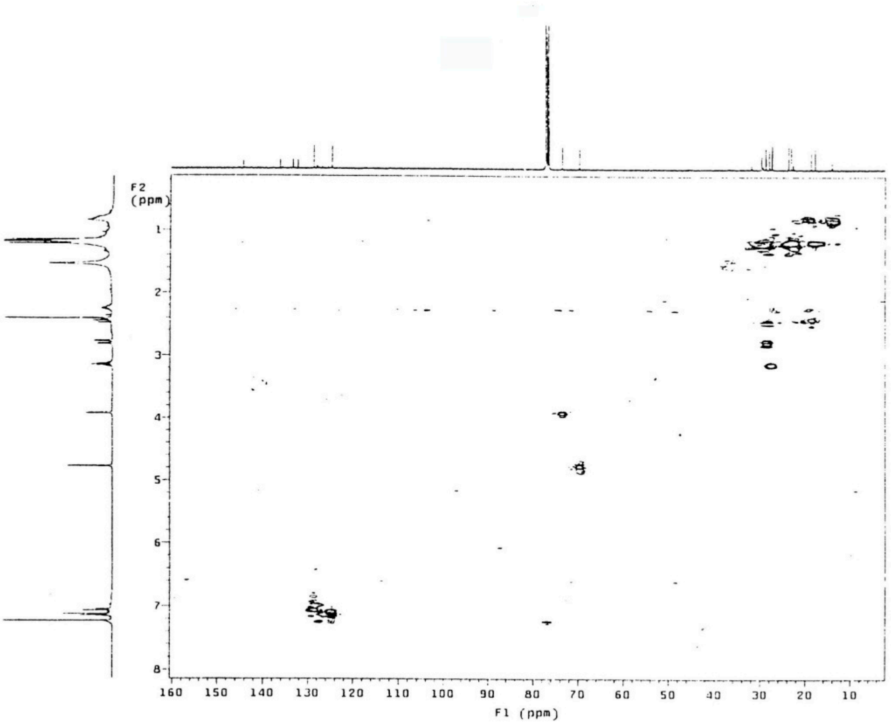

Figure S16. HMQC spectrum of compound **6**.

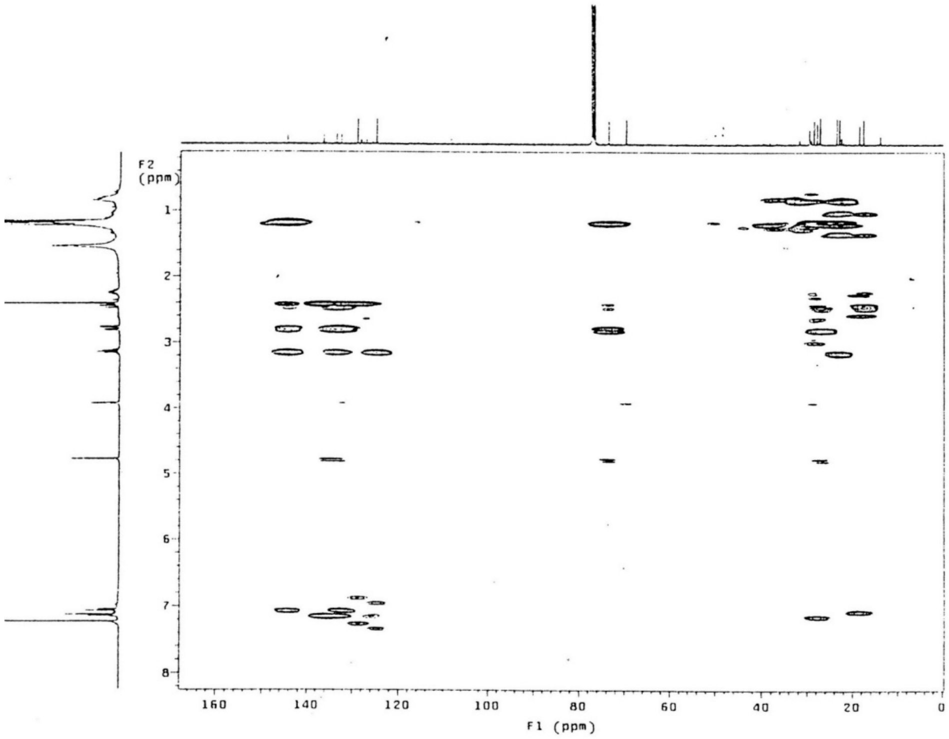

Figure S17. HMBC spectrum of compound **6**.

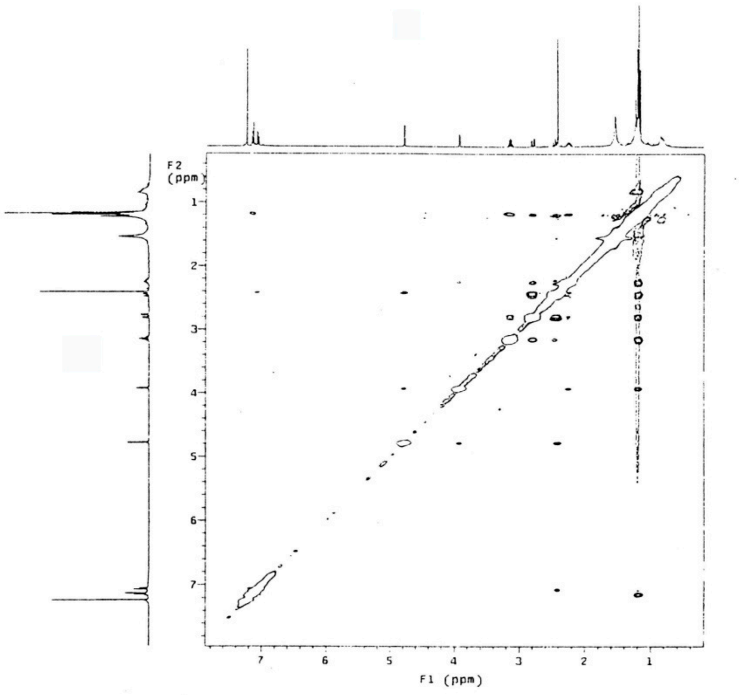

Figure S18. NOESY spectrum of compound **6**.

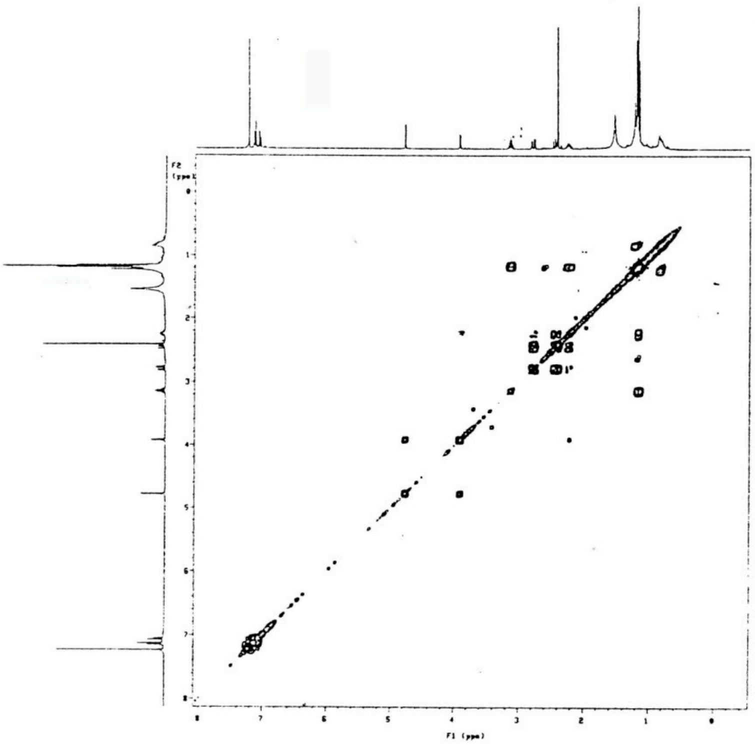

Figure S19. ^1H-^1H COSY spectrum of compound **6**.

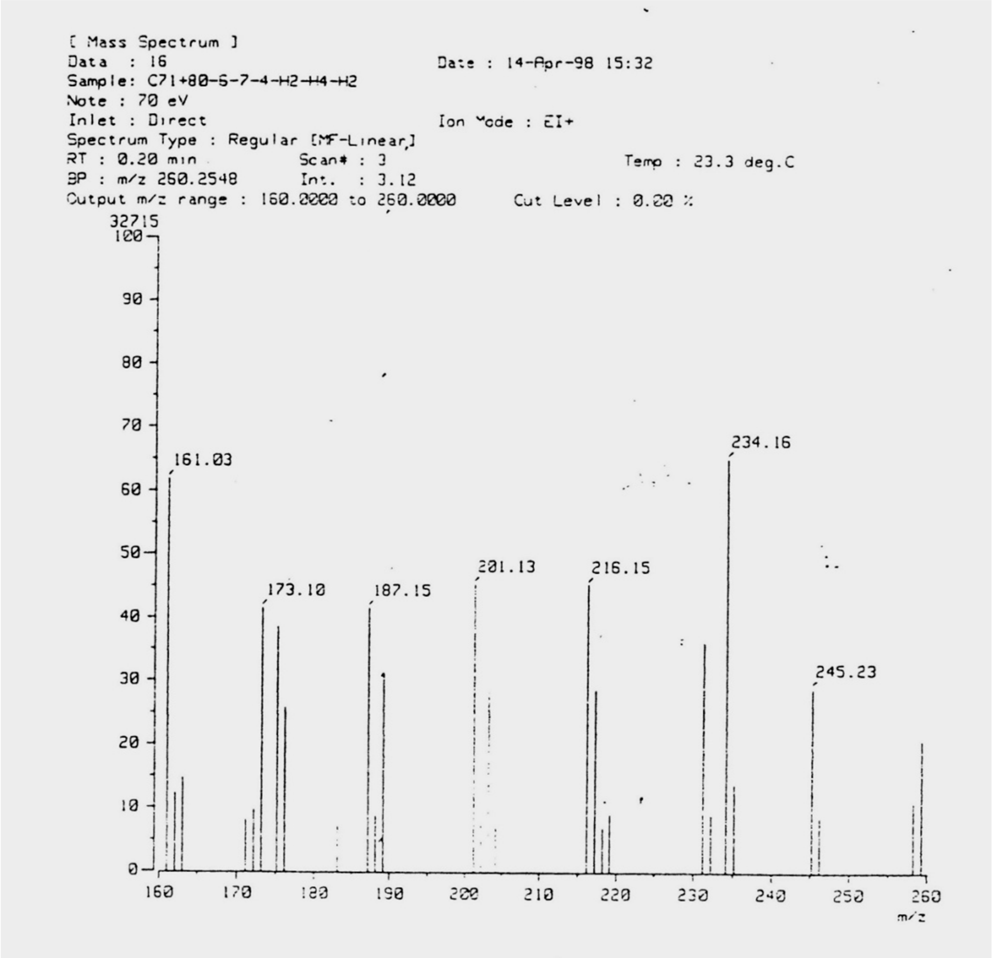

Figure S20. Mass spectrum of compound **6**.
